# Targeting Orphan Nuclear Receptors NR4As for Energy Homeostasis and Diabetes

**DOI:** 10.3389/fphar.2020.587457

**Published:** 2020-11-27

**Authors:** Chenyang Zhang, Bin Zhang, Xuelian Zhang, Guibo Sun, Xiaobo Sun

**Affiliations:** ^1^ Beijing Key Laboratory of Innovative Drug Discovery of Traditional Chinese Medicine (Natural Medicine) and Translational Medicine, Institute of Medicinal Plant Development, Peking Union Medical College and Chinese Academy of Medical Sciences, Beijing, China; ^2^ Key Laboratory of Bioactive Substances and Resource Utilization of Chinese Herbal Medicine, Ministry of Education, Beijing, China; ^3^ Key Laboratory of Efficacy Evaluation of Chinese Medicine against Glycolipid Metabolic Disorders, State Administration of Traditional Chinese Medicine, Beijing, China; ^4^ Key Laboratory of New Drug Discovery Based on Classic Chinese Medicine Prescription, Chinese Academy of Medical Sciences, Beijing, China

**Keywords:** orphan nuclear receptors, NR4As, energy homeostasis, metabolism, diabetes

## Abstract

Orphan nuclear receptors are important members of the nuclear receptor family and may regulate cell proliferation, metabolism, differentiation, and apoptosis. NR4As, a subfamily of orphan nuclear receptors, have been reported to play key roles in carbohydrate and lipid metabolism and energy homeostasis. Popularity of obesity has resulted in a series of metabolic diseases such as diabetes and its complications. While imbalance of energy intake and expenditure is the main cause of obesity, the concrete mechanism of obesity has not been fully understood. It has been reported that NR4As have significant regulatory effects on energy homeostasis and diabetes and are expected to become new targets for discovering drugs for metabolic syndrome. A number of studies have demonstrated that abnormalities in metabolism induced by altered levels of NR4As may contribute to numerous diseases, such as chronic inflammation, tumorigenesis, diabetes and its complications, atherosclerosis, and other cardiovascular diseases. However, systematic reviews focusing on the roles of NR4As in mediating energy homeostasis and diabetes remain limited. Therefore, this article reviews the structure and regulation of NR4As and their critical function in energy homeostasis and diabetes, as well as small molecules that may regulate NR4As. Our work is aimed at providing valuable support for the research and development of drugs targeting NR4As for the treatment of obesity and related metabolic diseases.

## Introduction

Obesity has been increasing in recent years (Heymsfield and Wadden, 2017). It is associated with insulin resistance, type 2 diabetes mellitus (T2DM), and its complications (Malone and Hansen, 2019). Correlated to an increase in food intake and a reduction in energy expenditure, obesity mainly results from energy imbalance (Spiegelman and Flier, 2001). In maintaining body weight, complex physiological processes incorporating both central and peripheral procedures are involved in achieving equilibrium of energy balance, namely, homeostasis (Narayanaswami and Dwoskin, 2017). It has been reported that, in regulating energy homeostasis, satiety, hunger, and adiposity signals derived from the periphery are incorporated with hypothalamus and brainstem, which contain distinct neuronal circuits and signaling molecules, in order to modulate feeding and metabolism (Blundell, 1991). A majority of anti-obesity drugs already used in clinic and those in development function in central mechanisms. However, owing to counterregulation, current pharmacological monotherapies finally develop into drug resistance (Ng et al., 2014). Although combination therapy may improve clinical outcomes, the occurrence of adverse effects may also increase, which in turn can cause numerous withdrawals of anti-obesity drugs from the market. Therefore, it is significant to research in the molecular mechanisms of obesity and the pathogenesis of T2DM, which can provide new therapeutic targets and strategies for the treatment of such diseases.

The nuclear receptor family plays an indispensable part in the survival of multicellular organisms. This family comprises a series of members as transcription factors that regulate the transcription and translation of various genes and participate in different biological processes (Mangelsdorf et al., 1995; Evans, 2005). The human nuclear receptor family has 48 members. Their transcriptional activity can be activated by direct binding of small molecules to the ligand-binding domain, thereby regulating the expression of some genes involved in development, endocrine signaling, and metabolism (Benoit et al., 2006). A total of 25 receptors in the nuclear receptor family are called orphan nuclear receptors because their natural ligands have not been discovered yet (Germain et al., 2006). The orphan nuclear receptors may promote or inhibit some gene expression or may be regulated by other signal transduction pathways through functional regulation of their transcriptional activation region. The transcriptional regulatory activity of these orphan receptors can be activated by specific coactivators at their DNA activation sites, subsequently leading to the activation or inhibition of target genes (Giguere, 2008). In recent years, it has been found that the orphan nuclear receptor 4A family (NR4As) can not only regulate immunity, inflammation, and tumor (Mohan et al., 2012; Rodriguez-Calvo et al., 2017), but also play key roles in maintaining energy homeostasis in the body (Close et al., 2013). The NR4A family comprises three members: NR4A1 (NUR77), NR4A2 (NURR1), and NR4A3 (NOR-1) (Nuclear Receptors Nomenclature, 1999). Members of this family have tissue-specific expression and exert different biological functions. In cytoplasm, this family can directly or indirectly affect the activity of mitochondria. In nucleus, the NR4A family interacts with other coactivators in order to regulate gene expression. Studies have confirmed that the NR4A family significantly regulates the expression of energy metabolism-related genes and other physiological processes. In the past few years, many new target genes that are regulated by the NR4A family and that exert important biological functions have been identified.

Energy imbalance is regarded as a main cause of obesity, which may result in diabetes and its complications, cardiovascular diseases, cancer, and other metabolic diseases. While NR4As are reported to regulate energy homeostasis, there are few articles that summarize and introduce the interaction between them and diabetes, as well as small molecules that may regulate NR4As. Therefore, we review the structure and regulation of the NR4A family and their roles in energy homeostasis and diabetes and its complications, as well as small molecules that may affect NR4As. Our work provides a reference for further research and development of candidate drugs targeting NR4As for the treatment of metabolic diseases.

## NR4A Structure and Function

### Structure and Regulation of NR4As

**FIGURE 1:**
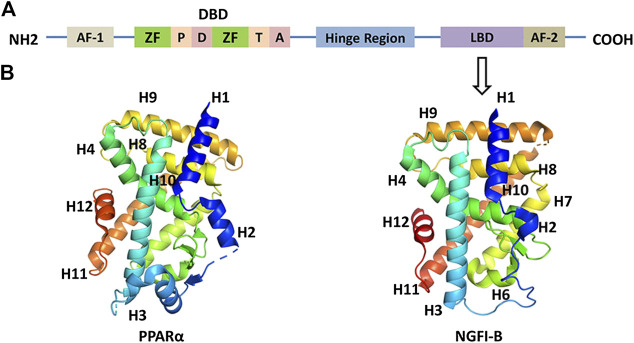
**(A)** Common structure of nuclear receptors. ZF: zinc finger; P: P box; D: D box; T: T box; A: A box; DBD: DNA-binding domain; LBD: ligand-binding domain. **(B)** 3-dimensional structure of LBD of PPARα (PDB code 3VI8) and NGFI-B (PDB code 1YJE). Helix 12 is shown in red.

Nuclear receptors are a family of ligand-dependent transcription factor superfamily which can bind to steroid hormones, vitamins, fat-soluble hormones, fatty acids, or other intracellular signaling molecules to regulate transcriptional responses. Nuclear receptor research began in the 1960s when Elwood V. Jensen of the University of Chicago discovered that steroid hormones regulate nuclear receptors, but he had limited knowledge of nuclear receptors including their structure and regulatory mechanisms (Toft et al., 1967). In 1985, Hollenberg and colleagues identified the first nuclear receptor and successfully cloned the full-length cDNA of human glucocorticoid receptor gene (Hollenberg et al., 1985). Then, Pierre Chambon cloned cDNA sequence of estrogen receptor α. Analysis of the sequences of these two genes revealed that certain regional structures are homologous to the oncogene v-erb-a, further confirming that the v-erb intracellular homologue is a thyroid hormone receptor (Green and Chambon, 1985). The above results demonstrate that the ligands of nuclear receptors include not only steroid receptors, but also mineralocorticoid receptors, progesterone receptors, and androgen receptors. It has been found that these receptors’ sequences are similar. They all include three functional domains: the ligand-binding domain (LBD), the DNA-binding domain (DBD), and the activation domain (shown in Figure 1A). AF-1 region, also known as the A/B region, is an N-terminal nonligand-dependent transcriptional activation region. The length and sequence of A/B regions of different nuclear receptors are quite different, revealing that this region is poorly conserved. This region can be used as a target not only for posttranscriptional modification, but also for interaction with coactivators or other transcription factors (Wang et al., 2003). DBD region, also known as the C region, is a highly conserved region of nuclear receptors. Nuclear receptors are bound by domain-specific DNA sequence (hormone response elements). The C region contains two highly conserved zinc finger structures consisting of four cysteines and zinc ions at the central site. Therefore, these two zinc finger structures are the basis for nuclear receptor targeting to bind hormone response elements. The zinc finger structure of the DBD region comprises a plurality of sequence elements such as P, D, T, and A sequences, which function by binding to the DNA backbone and its residues. For example, the P sequence is located between the two cysteines behind the first zinc finger structure, regulating the specificity of the nuclear receptor DNA-binding element (Schwabe and Rhodes, 1991). A single amino acid change in P box can cause the selection of DNA response elements by estrogen receptor and glucocorticoid receptor. The Hinge region, also known as the D region, is located between the DBD and the LBD. This region can rotate the DBD and form a different conformation without steric hindrance with the LBD, which is closely related to the nuclear location of the nuclear receptor. LBD area, also known as the E area, located at the C end, mediates the function of ligand binding and dimerization (shown in Figure 1B). This region contains four distinct but functionally related curved structures; its secondary structure consists of 12 α-helices, the last of which is AF-2 that mediates ligand-dependent transactivation and coactivator recruitment (Benoit et al., 2004). Many nuclear receptors have the activity of transcription factors under the regulation of their ligands, regulating biological processes including metabolism and energy balance. Therefore, nuclear receptors have become an emerging target for the treatment of certain diseases.

The orphan nuclear receptor 4A family is a group of nuclear receptors. In 1988, Milbrandt J identified the nucleotide amino acid sequence of NGFR-B in the search for growth factor-regulated protein factors (Milbrandt, 1988). In the same year, Hazel discovered the NUR77, namely, NR4A1 gene, when using growth factors to stimulate 3T3 fibroblasts in resting mice (Hazel et al., 1988). In 1992, Law SW identified NURR1 (NR4A2), a new brain-specific transcription factor (Law et al., 1992). Two years later, NOR-1 (NR4A3), a novel thyroid/steroid receptor superfamily gene, was cloned from cultured rat neuronal cells by Ohkura N et al. from Japan (Ohkura et al., 1994). Accordingly, NUR77, NURR1, and NOR-1 compose a new family of nuclear receptors, namely, nuclear receptor 4 subfamily A group, NR4As, which have the same structure as the nuclear receptors. The three members of the NR4A family share a high degree of homology and a common structure, including a ligand-independent activation-function-1 transactivation domain in the N-terminal region, a DNA-binding domain consisting of two zinc fingers, and a ligand-dependent AF-2 transactivation domain in their C-terminal region (Saucedo-Cardenas et al., 1997). However, X-ray crystallography studies show that NR4As encode an atypical LBD, which contains tightly packed, bulky hydrophobic residues rather than ligand-binding pockets found in other nuclear receptors, which are critical for the recruitment of small lipophilic molecules (Maruyama et al., 1998). Therefore, they are dubbed orphan receptors due to lack of natural ligands (Kliewer et al., 1999).

### DNA-Binding Activity of NR4As

The changes in physical factors induce expression of NR4As, which could regulate transcription of their target genes through directly binding to specific sequence sites in their promoter region. In particular, NR4As not only bind as monomers to a DNA-specific octamer sequence (AAAGGTCA), namely, NGFI-B-response element, but also bind as homodimers or NR4A-heterodimers to NGFI-B-response element palindromic sequences (TGATATTTACCTC CAAAATCCA), also known as the Nur-response element (Wilson et al., 1991; Wilson et al., 1993; Philips et al., 1997). In addition, except NR4A3, both NR4A1 and NR4A2 could bind as heterodimers with retinoid X receptors to target DR5 elements (GGT​TCA​CCG​AAA​GGT​CA) (Perlmann and Jansson, 1995; Zetterstrom et al., 1996). Thus, NR4A receptors can bind to specific response elements and cofactors in order to regulate gene expression in key cellular functions, including inflammation, proliferation, and cell survival (Herring et al., 2019).

### Diseases Related to NR4As

Accumulating evidence has shown that NR4As play crucial roles in regulating various biological processes, including cell proliferation and differentiation, organ development, and immune homeostasis (Maxwell and Muscat, 2006). NR4A receptors are also found to participate in the pathological mechanisms of numerous diseases, including neuronal disease, inflammation, cancer, and cardiovascular diseases. NR4As have been widely verified to mediate relevant neuronal functions (Hawk and Abel, 2011). It was found that NR4As are closely associated with dopamine neuron differentiation and survival. Therefore, these orphan receptors might be potential targets for treating Parkinson’s disease (Paillasse and de Medina, 2015). In addition, NR4A3 has been reported to regulate depressive behavior and nicotine addiction in patients with mental illness (Novak et al., 2010; Schaffer et al., 2010). Furthermore, NR4As play a dual role in cancer, showing both pro-oncogenic and tumor suppressor-like activities (Safe et al., 2014). NR4A1 is found to be overexpressed in numerous cancers, leading to increased proliferation and survival in these cancer cells via upregulating some target genes, including cyclin D2, E2F1, and survivin. Oppositely, NR4A1 knockdown decreases cancer cellular rate and angiogenesis through activating the apoptotic pathways. High expression of NR4A2 is closely associated with cancer progression and promoting cancer cell migration (Ranhotra, 2015). Little is known about the functions of NR4A3 in cancer, though some crucial findings have been reported. NR4A3 inhibited lymphomagenesis through the upregulation of proapoptotic genes, including BCL2 antagonist/killer 1, Puma, and BCL2 interacting killer (Reynolds et al., 2016; Deutsch et al., 2017). Certainly, the key roles of NR4As in immune response cannot be neglected. These three receptors are important for the maintenance of Treg cells and CD81 T cell development (Sekiya et al., 2013; Nowyhed et al., 2015); they also participate in autoimmunity and protect against infections. Studies have shown that NR4A1 knockout caused macrophage differentiation towards M1 phenotype, which may promote inflammatory response (Chao et al., 2013). Conversely, NR4A2 mediates the gene expression of M2 phenotype and confers protection against sepsis (Mahajan et al., 2015). Increasing evidence suggests that NR4As indisputably exert a panoply of essential roles in regulating multiple biological processes. However, a summary of the metabolic roles governed by NR4A members remains incomprehensive. Therefore, this review mainly focuses on the potential roles of NR4A in regulating energy homeostasis and diabetes, as well as on potential drugs in regulating NR4As.

## Potential Roles of NR4As in Energy Homeostasis

### NR4As and Glucose Metabolism

**FIGURE 2:**
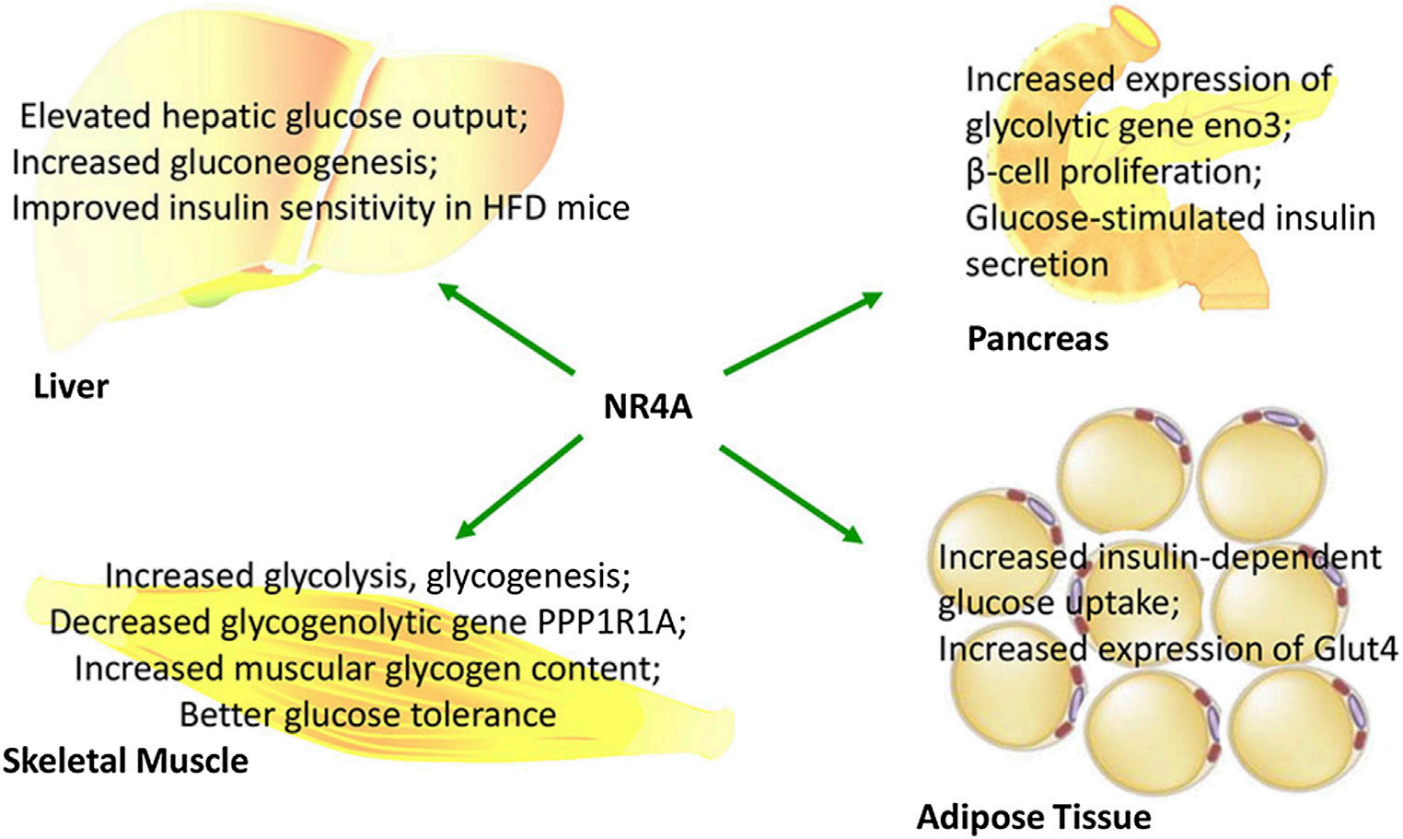
NR4As function in glucose metabolism and insulin activity in a tissue-specific manner.

Increasing evidence has indicated that NR4A members are important regulators of glucose utilization, gluconeogenesis, and glycogenesis (Close et al., 2013). While NR4As promote glucose utilization in muscles, adipose tissue, and islets, NR4A1 can also promote glucose production in the liver. However, NR4As exert their roles in different tissues in independent ways. To date, no research has reported their effect on cross-talk between liver, muscle, or adipose in glucose metabolism. Therefore, the impact of NR4As on whole-body glucose homeostasis remains to be further researched. The roles of NR4As in regulating glucose metabolism are summarized in Figure 2.

### Glucose Utilization

Skeletal muscle is the largest glucose-utilizing in the human body. Therefore, abnormal expression of NR4As in skeletal muscle may disturb glucose homeostasis. Knockout of NR4A1 in skeletal muscle not only decreased expression of *GLUT4*, an insulin-sensitive glucose transporter, but also reduced expression of a series of genes involved in glycolysis, including phosphofructokinase (*PFK*), phosphoglycerate mutase 2 (*PGAM2*), and glycerophosphate shuttle (Chao et al., 2007). Some of these effects may be reproduced on NR4A3. Overexpression of NR4A3 in skeletal muscle could upregulate glycolytic genes including *PFK1, PGAM2*, hexokinase 1 (*HK1*), enolase 3 (*ENO3*), glyceraldehyde-3-phosphate dehydrogenase (*GAPDH*), and pyruvate kinase isozyme type M (*PKM*). In addition, glycogenic genes such as *GLUT4, HK2*, and glycogen synthase 1 (*GYS1*) were also upregulated by NR4A3 overexpression. Moreover, the glycogenolytic gene protein phosphatase 1 regulatory subunit A (*PPP1R1A*) was repressed, which was followed by increased muscular glycogen content (Pearen et al., 2008). Collectively, NR4A1 and NR4A3 promote both glycolysis and glycogenesis in skeletal muscle.

Apart from muscle, NR4As also play a pivotal role in regulating glucose utilization in adipose tissue. NR4A1 and NR4A3 in adipocytes may activate insulin signaling pathways by stimulating insulin-dependent glucose uptake (Fu et al., 2007). Overexpressing NR4A3 increased the expression of *GLUT4* and its translocation to the plasma membrane, whereas knocking down NR4A3 impaired glucose uptake in adipocytes (Fu et al., 2007).

Meanwhile, in the pancreatic β-cells, glycolytic genes may also be regulated by NR4A1 and NR4A3, which modulate glucose utilization and insulin secretion. Treating β-cells with palmitate or oleate may induce the expression of NR4A1 (Roche et al., 1999). In the MIN-6 cell line, culturing with palmitate increased NR4A1 expression, which subsequently increased the expression of the glycolytic gene *ENO3* (Briand et al., 2012). Similar results were found in the 832/13 INS-1 β-cell line, where loss of NR4A1 or NR4A3 decreased the expression of *ENO1* (Maris et al., 2011).

### Glucose Production

In patients with type 2 diabetes, elevated output of hepatic glucose is an important factor leading to both fasting and postprandial hyperglycemia (DeFronzo, 1992). In mouse hepatocytes, NR4As can be rapidly induced by cAMP, and in mouse liver, they can be prompted by glucagon or conjugated linoleic acid trans-10, cis-12-CLA (Pei et al., 2006). Overexpression of NR4As in liver induced the expression of genes involved in gluconeogenesis, including glucose-6-phosphatase (*G6PC*), fructose-1, 6-bisphosphatase 1 (*FBP1*), *ENO*3, and Slc2a2 (Pei et al., 2006). In addition, enhanced hepatic glucose output and elevated fasting glucose levels were observed in mice with NR4A1 liver-specific overexpression, and it was suggested that NR4A1 might govern gluconeogenesis through an alternative pathway except for cAMP-CREB (Berriel Diaz et al., 2006). Similarly, mice treated with NR4A1 agonist, cytosporone B, also showed increased blood glucose levels and induced expression of genes mentioned above (Zhan et al., 2012). In contrast, after silencing NR4A1 in db/db mice, lower blood glucose levels and reduced expression of *FBP1* and Slc2a2 were observed (Close et al., 2013). In NR4A1 knockout mice fed with a high-fat diet, expression of liver glycolytic genes such as *ENO3*, bisphosphoglycerate mutase (*BPGM*), and phosphoglycerate kinase 1 (*PGK1*), as well as gluconeogenic genes *FBP1* and peroxisome proliferator-activated receptor γ coactivator-1α (*PGC1α*), was upregulated, while expression of *G6PC* was downregulated (Chao et al., 2009). Moreover, hepatocellular carcinoma can be suppressed by NR4A1 by attenuating phosphoenolpyruvate carboxykinase (*PEPCK*) sumoylation, which switches glucose metabolism towards gluconeogenesis (Bian et al., 2017). Overall, these data indicate that NR4A1 plays a central role in regulating gluconeogenesis in liver and hepatocytes.

### NR4As and Lipid Metabolism

**FIGURE 3:**
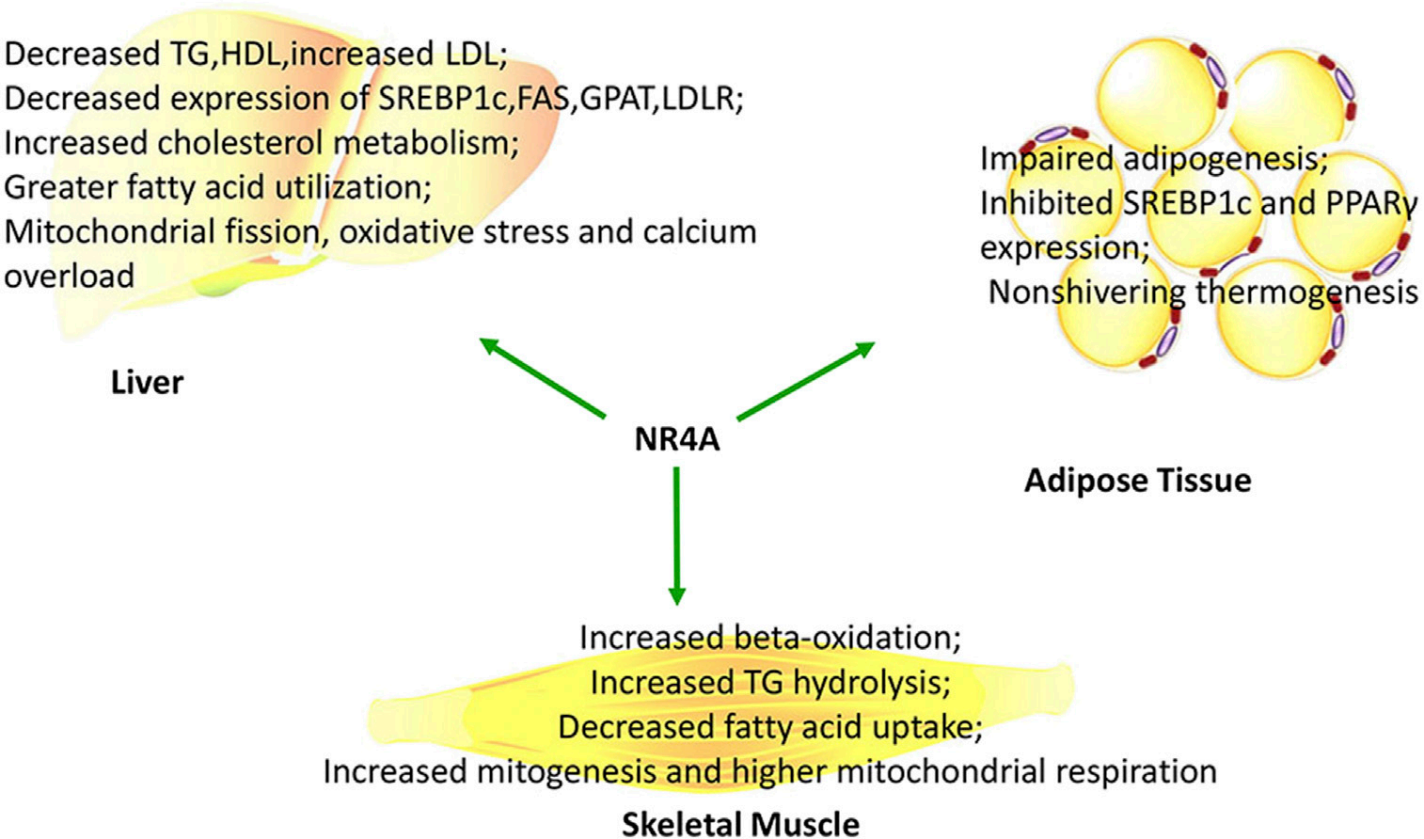
NR4As influence lipid metabolism and mitochondrial activity in a tissue-specific manner.

NR4A1 participates in liver lipid metabolism. Abnormal liver expression of NR4A1 is implicated in fat metabolism, cholesterol metabolism, hepatic steatosis, and many other pathophysiological processes (summarized in Figure 3). Full-body NR4A1 knockout mice fed with a high-fat diet showed elevated liver triglycerides, cholesterol, and exacerbated hepatic steatosis (Chao et al., 2009). Knockout of NR4A1 led to increased even-chain acyl carnitine species and elevated expression of hepatic lipoprotein lipase (*LPL*), as well as decreased expression of hepatic enoyl-CoA hydratase (*ECHS*) and 3-hydroxyacyl-CoA dehydrogenase (*EHHADH*) (Chao et al., 2009). Adenovirus-mediated liver-specific NR4A1 overexpression mice had decreased circulating triglyceride levels and high-density lipoprotein (HDL) levels but increased low-density lipoprotein (LDL) levels (Pols et al., 2008). This was correlated with significantly decreased expression of sterol regulatory element-binding protein 1c (*SREBP1c*), which further suppressed its downstream genes such as stearoyl-CoA desaturase 1 (*SCD1*), fatty acid synthase (*FAS*), mitochondrial glycerol-3-phosphate acyltransferase (*GPAT*), and low-density lipoprotein receptor (*LDLR*) (Pols et al., 2008). It was also reported that NR4A1 can regulate hepatic cholesterol metabolism by suppressing expression of LDLR and 3-hydroxy- 3-methylglutaryl-coenzyme A reductase (*HMGCR*) (Zhang et al., 2012). However, elevated cholesterol levels may in turn upregulate NR4A1 expression (Kudo et al., 2004). NR4A1 or NR4A2 overexpression increased carnitine palmitoyl transferase 1a (*CPT1a*) in HepG2 cells, which permitted greater mitochondrial translocation of fatty acids (Ishizawa et al., 2012). These above-mentioned data demonstrate that NR4A1 and NR4A2 cause greater liver fatty acid utilization. While NR4A1 suppressed lipogenesis through suppressing *SREBP1c, LDL* increased owing to decreased expression of *LDLR*.

Body-fat content and insulin sensitivity may influence NR4A1 expression; therefore, muscles of obese men have much lower NR4A1 expression than that of lean men (Sinha et al., 2002). High-fat diet-fed NR4A1-null mice showed elevated intramuscular triglycerides and diacylglycerol levels, possibly due to greater fatty acid uptake and decreased oxidation, which was accompanied by increased expression of acylcarnitine and lipoprotein lipase (Chao et al., 2009). Similarly, knocking down NR4A1 in C2C12 muscle cells decreased hydrolysis of triglycerides, which corresponded to decreased expression of CD36, *CPT1*, adiponectin receptor 2, adenosine 5’-monophosphate- (AMP-) activated protein kinase gamma 3 (*AMPKγ3*), uncoupling protein 3 (*UCP3*), and caveolin-3 (Maxwell et al., 2005). Meanwhile, SREBP1c, a major regulator of lipogenesis, was induced in NR4A1 knockdown, thereby indicating the role of NR4A1 in triglyceride accumulation (Maxwell et al., 2005). In addition, overexpression of NR4A3 can increase β-oxidation in muscles. This corresponded to increased expression of carnitine acetyltransferase (*CRAT*), long-chain fatty acyl-CoA synthetase (*ACSL*), acetaldehyde dehydrogenase (*ACDH*), enyol-CoA hydratase (*ECH*), and 3-ketoacyl-CoA thiolase (*3-KCT*) (Pearen et al., 2013). However, loss of NR4A3 in C2C12 cells impaired palmitate oxidation and increased anaerobic glycolysis (Pearen et al., 2013). This was in accordance with decreased expression of lipin1α, *PGC1*, and pyruvate dehydrogenase phosphatase 1 (*PDP1*) (Pearen et al., 2008). Conversely, formoterol, an activator of β-adrenergic pathway, may activate NR4A3, which in turn enhances genes involved in oxidative metabolism (Pearen et al., 2008). These data indicate that NR4A1 and NR4A3 are essential for lipid metabolism in muscles.

NR4As are highly upregulated during obesity and are significantly downregulated after fat loss (Veum et al., 2012). When fed with high-fat diet for 4 months, female NR4A1 deficient mice showed increased body weight and fat mass compared to males, indicating that NR4A1 modulates lipid metabolism in adipose tissue in a gender-specific manner (Perez-Sieira et al., 2013). Stimulation with β-adrenergic and fasting induced the expression of NR4A1, which can directly bind to promoter of peroxisome proliferator-activated receptor γ (*PPARγ*) and subsequently reduce its expression in white adipose tissue (Duszka et al., 2013). NR4A1 induced p53 and GATA2 expression, which subsequently inhibited expression of *SREBP1c* and impaired adipogenesis (Qin et al., 2018). However, overexpression of NR4A3 in adipocytes enhanced insulin-stimulated glucose transport and insulin signaling, while transgenic mice with adipose tissue-specific NR4A3 overexpression displayed no difference in lipolysis and impaired insulin signaling (Walton et al., 2016). Meanwhile, reports on the role of NR4As in adipocytes also conflict with each other. Au et al. demonstrated that loss of NR4A1 in 3T3-L1 preadipocytes had no effect on adipogenesis or lipid accumulation (Au et al., 2008), which was opposed to Jung et al.’s report, which held a view that inhibiting NR4A1 activity may decrease lipid accumulation in 3T3-L1 cells (Jung et al., 2019). In addition, a follow-up study demonstrated that NR4A1 was indispensable for the function of adipocyte progenitor (Zhang et al., 2018). Our knowledge on NR4A and lipid metabolism is notwithstanding growing. However, how NR4As may influence lipid metabolism still needs to be further investigated in the future.

### NR4As and Mitochondrial Function

Diverse studies have reported that NR4As may regulate mitochondrial production and fuel oxidation (summarized in Figure 3). This incorporates transcriptional regulation of β-oxidation, tricarboxylic acid cycle, and the electron transport chain. For example, knocking down NR4A1 decreased mitochondrial fission, oxidative stress, and calcium overload in nonalcoholic fatty liver mediated by high-fat diet (Zhou et al., 2018). However, primary hepatocytes manifested decreased mitochondrial respiration when exposed to excess palmitate, due to upregulation of NR4A1.

Opposed to the liver, overexpressing NR4A1 in C2C12 muscle cells induced increased mitochondria content and activity, whereas knocking down NR4A1 in C2C12 cells and primary muscle cells displayed decreased uncoupling protein 3 expression (Maxwell et al., 2005). Similar to these cells, animal muscles overexpressing NR4A1 showed improved muscle strength because of greater mitochondrial DNA concentrations, which implied increased mitogenesis and higher mitochondrial respiration (Chao et al., 2012). An increased NR4A1 and NR4A3 expression was observed in soleus and ETS-domain lacking muscle after mice trained for running potential. Meanwhile, expressions of NR4A targets CD36 and UCP3, as well as the electron transport chain components Complexes 1–5, were also elevated (Kawasaki et al., 2009). Similarly, NR4A3 overexpression in muscles increased tricarboxylic acid cycle flux by increasing oxoglutarate dehydrogenase, isocitrate dehydrogenase 3 (*IDH3*), fumarate hydratase (*FH*), succinate dehydrogenase B (*SDHB*), and malate dehydrogenase (*MDH*) expression (Stephenson et al., 2012; Pearen et al., 2013). Elevated contents of mitochondrial electron transport chain components were also observed, including SDHB, ATP synthase F1α (*ATP5a1*), ubiquinol-cytochrome c reductase core protein 2 (*UQCRC2*), cytochrome c oxidase 1 (*COX1*), and NADH:ubiquinone oxidoreductase b8 (*NDUFB8*) (Stephenson et al., 2012). Together, these data clearly demonstrate that NR4A1 and NR4A3 are indispensable for mitochondrial content and fuel oxidation in muscles.

In addition to the muscle, NR4A1 is also involved in the nonshivering thermogenesis of brown adipose tissue by directly binding to the promoter of *UCP3* and inducing its expression (Kanzleiter et al., 2005). NR4A1 could be induced by β-adrenergic signaling and knockdown of NR4A1 inhibited *UCP1* gene transcription *in vitro* (Kanzleiter et al., 2005). It was also reported that NR4A3 also could bind to the promoter of *UCP1* and induce its expression (Kumar et al., 2008). These data collectively demonstrate that NR4As play essential roles in mitochondrial genesis and uncoupling in brown adipose tissue.

## Potential Roles of NR4As in Diabetes

### Insulin Secretion

NR4As were highly expressed in β-cells. NR4A1 promoter was reported to be hypermethylated in patients and in a mouse model of T2DM (Chen et al., 2016). However, when treated with aurintricarboxylic acid, a DNA methyltransferase 1 inhibitor, T2DM mice presented increased NR4A1 expression in pancreatic β-cells and decreased blood glucose (Kudo et al., 2004; Chen et al., 2016). In pancreatic β-cells, NR4A1 can be induced by glucose and saturated fatty acids (Susini et al., 1998; Roche et al., 1999), which subsequently regulates cell proliferation and insulin secretion (shown in Figure 2) (Briand et al., 2012). Knockout of NR4A1 decreased β-cell density in the islets. Moreover, NR4A1 and NR4A3 can induce β-cell proliferation after stimulated by homeodomain transcription factor NK6 homeobox 1 in mouse pancreatic islets (Tessem et al., 2014). Their loss of function in 832/13 INS-1 β-cells impaired mitochondrial respiration and significantly decreased tricarboxylic acid cycle and electron transport chain components *IDH3* and *SDHB*. As a result, decreased NADH and FADH_2_ cycling, decreased ATP levels, and ultimately decreased glucose-stimulated insulin secretion were found in NR4A1 and NR4A3 knockout β-cells (Reynolds et al., 2016; Close et al., 2020). However, in the clonal MIN6 β-cell line, NR4A1 overexpression impaired glucose-stimulated insulin secretion owing to decreased expression of transcription factors regulating insulin gene transcription, including macrophage-activating factor b-ZIP transcription factor A (*MAFA*), pancreatic and duodenal homeobox 1 (*PDX-1*), and NeuroD1 (Briand et al., 2012). Furthermore, NR4A1 and transcription factor FoxO1 reduced insulin secretion in human pancreatic β-cells through protein–protein interaction, thereby controlling glucose homeostasis (Briand et al., 2012). Moreover, NR4A1 and NR4A2 might influence insulin secretion by disrupting zinc homeostasis, through which they regulate cation homeostasis by regulating zinc transporters Slc30a1 and Slc39a8 (Rutter, 2010). The conflicting results of NR4As may be due to different cell lines used in research. Therefore, the role of NR4As in regulating insulin secretion remains to be further demonstrated.

### Insulin Resistance

Studies have demonstrated conflicting findings when comparing liver insulin sensitivity in full-body NR4A1 knockout mice. When fed with chow diet, NR4A1 knockout mice showed improved insulin sensitivity, whereas, when fed with a high-fat diet, they showed impaired glucose tolerance and elevated circulating insulin levels (Chao et al., 2009). Chao et al. also demonstrated that the latter showed muscular insulin resistance by decreased levels of insulin receptor phosphorylation and glycolytic expression. However, G6Pase, which allows glucose to be released from hepatocytes, was downregulated in both conditions (Chao et al., 2009). It is important to note, however, that these findings need to be further validated. A recent study demonstrated that in N-terminal domain only truncated NR4A1 could interact with and stabilize hypoxia inducible factor 1α, which caused some unexpected nongenomic functions mistaken for genomic effects (Herring et al., 2019). Reports have demonstrated that muscle-specific NR4A3 transgenic mice exhibit better glucose tolerance, but not insulin sensitivity compared with wild-type mice (Pearen et al., 2012). However, muscle-specific NR4A1 overexpression could not protect mice against diet-induced glucose intolerance (Chao et al., 2012). Therefore, NR4As expression in liver and muscle may influence whole-body glucose metabolism and insulin sensitivity (Fu et al., 2007), but their essential roles in regulating insulin sensitivity remain to be further researched.

### Small Molecules in Regulating NR4As

It has been found that cytosporone B and its related analogs bind to residue Tyr453 of NR4A1 LBD. Cytosporone B is an NR4A1 agonist that can induce hepatic gluconeogenesis in C57BL/6 mice, thereby increasing their blood glucose levels (Zhan et al., 2008). Ethyl [2,3,4-trimethoxy-6-(i-octanoyl) phenyl] acetate (TMPA) was found to bind to LBD area of NR4A1 at two sites. Arg515, Glu445, and Thr595 in the first site and Lys456, His372, Arg450, Tyr453, Leu492, and Val498 in the second site of the LBD were involved in the binding (Zhan et al., 2012). TMPA, an NR4A1 antagonist, decreased blood glucose and insulin levels in db/db mice and high-fat diet/STZ-treated mice. These effects were abolished in the absence of NR4A1, which proved that the effect of TMPA depended on NR4A1 (Zhan et al., 2012). In addition, hepatic gluconeogenesis in db/db mice could also be inhibited by TMPA as evidenced by nuclear export of free liver kinase B1 (*LKB1*), which in turn phosphorylates AMPKα and represses phosphoenolpyruvate carboxykinase (*PEPCK*) and *G6PC* expression (Zhan et al., 2012). It was also reported that 1,1-bis(3’-indolyl)-1-(pchlorophenyl)methane (C-DIM12), a synthetic small molecule, activated NR4A2 in a ligand-dependent manner. However, after knocking down NR4A2, its effect on poly(ADP-ribose) polymerase (*PARP*) cleavage and apoptosis in bladder cancer cells could be abolished (Inamoto et al., 2008). Recent studies have also identified several C-DIM analogs which could bind to the LBD area of NR4A1, suggesting that specific C-DIM compounds with small polar substituents on the phenyl ring could directly bind to the receptor and modulate NR4A transcriptional activity (Lee et al., 2014).

In addition, the anti-metabolite cancer drug, 6-mercaptopurine used to treat leukemia, could induce NR4A2 and NR4A3 through binding to AF-1 domain at the N-terminal of the latter; however, how it induced NR4A2 remains to be further researched (Ordentlich et al., 2003). Furthermore, several benzimidazole compounds, as well as a series of isoxazolopyridinone compounds, have high affinity for NR4A2 (Pearen and Muscat, 2010). Berberine, an oral antidiabetic drug, has been reported to activate AMPK and increase expression of hepatic fibroblast growth factor 21 via NR4A1 (Hirai et al., 2019). Prostaglandin A2 was also reported to enhance cellular insulin sensitivity via a mechanism involving NR4A3 (Zhu et al., 2013). Therefore, small molecules can also exhibit multiple beneficial effects on energy metabolism by targeting NR4As and are being thoroughly researched.

In addition to these drugs, numerous small molecules have been reported to regulate NR4As and metabolic diseases (shown in Supplementary Table S1). However, they may act on NR4As in an indirect way, and their exact mechanisms in regulating metabolism remain to be further elucidated. There are several small molecules that may regulate all three members of the NR4A family in treating metabolic diseases, which should be paid more attention because NR4As exert their roles in a tissue-specific manner. It should also be noted that using these small molecules may result in unexpected side effects.

## Conclusions

Obesity has become a 21st century epidemic, which may contribute to type 2 diabetes mellitus, cardiovascular diseases, cancer, and other health problems (Williams et al., 2015). There are many factors that may lead to excessive fat deposition in obesity, but unbalanced energy intake and consumption are widely considered to be the main reason. Controlling diet, exercise, and lifestyle changes are fundamental for the treatment of obesity, but medical care and bariatric surgery are becoming more significant (Gonzalez-Muniesa et al., 2017). Although bariatric surgery improved metabolism independent of weight loss, numerous side effects have also been found (Urdampilleta et al., 2012). Drugs on patients with obesity are only approved for use with dieting and exercise. None of them can be used on pregnant, nursing, or pediatric populations (Solas et al., 2016). The orphan nuclear receptor 4A family plays essential roles in inflammation, tumorigenesis, carbohydrate and lipid metabolism, energy homeostasis, diabetes, and other metabolic diseases, and some of these eventuate in a tissue-specific mechanism. NR4As act as critical pharmaceutical targets for various metabolic disorders. Overexpression or knockdown of NR4As can result in abnormal metabolism of organs or cell lines. Therefore, by studying their roles in regulating metabolism and determining their concrete mechanism, we will get more opportunities to develop medical treatments that protect against obesity and diabetic diseases.

Currently, numerous drugs are still being researched, and a large number of them have to be withdrawn because of their side effects, but the clinical demand is pushing researchers to find more effective and safe drugs to treat obesity and related complications. NR4As have been reported to regulate glucose production and utilization, lipid metabolism, and mitochondrial function as well as insulin secretion and insulin resistance. Therefore, this article reviews the structure and regulation of NR4As and roles of NR4As in energy homeostasis and diabetes, as well as small molecules that may regulate NR4As, aimed at providing further evidence for research into the mechanisms and therapeutic reagents of obesity and related metabolic diseases.

In summary, NR4As may promote glucose utilization in muscles, adipose tissue, and islets, while NR4A1 may also promote glucose production in liver. NR4As reduced lipid accumulation in muscles, but their exact roles in liver and adipose tissue remain to be further illustrated. NR4As were observed to increase content and activity of mitochondria in muscles and adipose tissue, but in the liver, there were contradictory results. The roles of NR4As in insulin secretion and insulin resistance were also different in different cell lines and mice models used for research. Currently, various studies have demonstrated how NR4As act on each organ or cell line, but none of them have demonstrated how NR4As maintain global energy homeostasis by regulating cross-talk between organs. TMPA was found to be an NR4A1 antagonist which could reduce blood glucose by reducing liver gluconeogenesis, whereas, acting as an NR4A1 agonist, cytosporone B increased blood glucose. Berberine, prostaglandin A2, and many other small molecules have also been reported to influence NR4As. However, how they act on NR4As and their exact mechanisms in regulating metabolism and diabetes remain unknown.

The current study is just in its infancy. There are still many problems, mainly pertaining to three aspects. First, while no research has identified endogenous ligands for NR4As, presumptive endogenous ligands, as well as pharmaceutical agonists and antagonists, have begun to be explored. Their ability to modulate function of NR4As and their effects on diverse tissues, as well as cross-talk between different tissues, need to be further explored. Furthermore, while numerous small molecules are reported to regulate NR4As, how they act on NR4As and their exact roles in regulating energy homeostasis and diabetes remain to be further demonstrated. Finally, it is widely known that activities of NR4A family members can be modulated by posttranslational modifications. However, greater circumscription of types and locations of the modifications, as well as their effects on obesity-related metabolic diseases such as diabetes, is required.

## Author Contributions

XS and GS conceived the research. CZ wrote the original draft. BZ revised the manuscript. CZ and XZ reviewed and edited the manuscript. All authors read and approved the final version of this manuscript. All authors have read and agreed to the published version of the manuscript.

## Funding

This study was supported by National Natural Science Foundation of China (Grant No. 81773936), the National Science Foundation for Young Scientists of China (Grant No. 81803803), Key Laboratory Project of CAMS (Grant No. 2018PT35030), National Key R&D Plan (No. 2017YFC1702504), and the Drug Innovation Major Project (Grant No. 2018ZX09711001–009).

## Conflict of Interest

The authors declare that the research was conducted in the absence of any commercial or financial relationships that could be construed as a potential conflict of interest.

## Abbreviations

**Abbreviations:** DBD, DNA-binding domain; G6PC, Glucose-6-phosphatase; LBD, Ligand-binding domain; PGC1, Peroxisome proliferator-activated receptor γ coactivator 1; T2DM, Type 2 diabetes mellitus; SREBP1, Sterol regulatory element-binding protein 1; UCP1, Uncoupling protein 1.
