# Supplementary material for: Targeting Orphan Nuclear Receptors NR4As for Energy Homeostasis and Diabetes
Source: Front Pharmacol. 2020 Nov 27;11:587457. doi: 10.3389/fphar.2020.587457 (PMC7728612; doi:10.3389/fphar.2020.587457)
Supplement: Supplementary file 1 [file Table1_v2.docx]

Supplementary Material

**Supplementary Table 1.** Summary of small molecules regulating NR4As in metabolic diseases.

|  | **NR4A1** | **NR4A2** | **NR4A3** |
| --- | --- | --- | --- |
| 1 | (+)-JQ1 compound (Fujiki et al., 2009) | (+)-JQ1 compound (Fujiki et al., 2009) | 15-Acetyldeoxynivalenol (Zhang et al., 2006) |
| 2 | 1-Methyl-4-phenylpyridinium (Fujiki et al., 2009) | 1,1-Bis(3’-indolyl)-1-(pchloro-phenyl) methane (C-DIM12) (Inamoto et al., 2008) | 1-Methyl-4-phenylpyridinium (Fujiki et al., 2009) |
| 3 | 2-(2-Amino-3-methoxyphenyl)-4H-1-benzopyran-4-one (Zhang et al., 2006) | 2-Methyl-2H-pyrazole-3-carb-oxylic acid (2-Methyl-4-o-tolylazophenyl)amide (Fujiki et al., 2009) | 2-(4-Morpholinyl-8-phenyl-4H-1- benzopyran-4-one (Zhang et al., 2006) |
| 4 | 2,2',4,4'-Tetrabromodiphenyl ether (Bongard et al., 2006) | 2-Methyl-4-isothiazolin-3-one (Zhang et al., 2015) | 2-Methyl-4-isothiazolin-3-one (Zhang et al., 2015) |
| 5 | 2-Methyl-4-isothiazolin-3-one (Zhang et al., 2015) | 3-(4-Methylphenylsulfonyl)-2-propenenitrile (Declerck et al., 2017) | 4-(5-Benzo(1,3)dioxol-5-yl-4-pyr- idin-2-yl-1H-imidazol-2-yl)benzamide (Declerck et al., 2017) |
| 6 | 7,8-Dihydro-7,8-dihydroxybe-nzo(a)pyrene 9,10-oxide (Huang et al., 2009) | 4-(5-Benzo(1,3)dioxol-5-yl-4- Pyridin-2-yl-1H-imidazol-2-yl)benzamide (Declerck et al., 2017) | 6-Mercaptopurine (Ordentlich et al., 2003) |
| 7 | 8-Bromo Cyclic Adenosine Monophosphate (Huang et al., 2009) | 6-Mercaptopurine (Ordentlich et al., 2003) | 8-Bromo Cyclic Adenosine Monophosphate (Huang et al., 2009) |
| 8 | Abrine (Gunes et al., 2009) | 7,8-Dihydro-7,8-dihydroxybe-nzo(a)pyrene 9,10-oxide (Kuhara et al., 2009) | Abrine (Gunes et al., 2009) |
| 9 | Acetaldehyde (Zhang et al., 2015) | 8-Bromo Cyclic Adenosine Monophosphate (Huang et al., 2009) | Arsenite (Declerck et al., 2017) |
| 10 | Acetaminophenv (Declerck et al., 2017) | Acetaldehyde (Zhang et al., 2015) | Asbestos (Zhang et al., 2015) |
| 11 | Acrolein (Zhang et al., 2015) | Afimoxifene (Zhang et al., 2015) | Ascorbic Acid (Declerck et al., 2017) |
| 12 | Afimoxifene (Huang et al., 2009) | Aflatoxin B1 (Fujiki et al., 2009) | Aspirin (Bongard et al., 2006) |
| 13 | Aflatoxin B1 (Fujiki et al., 2009) | Amiodarone (Bongard et al., 2006) | Atrazine (Zhang et al., 2006) |
| 14 | Arsenic Trioxide (Gunes et al., 2009) | Anacetrapib (Kuhara et al., 2009) | Benzene (Peyre et al., 2014) |
| 15 | Asbestos (Zhang et al., 2015) | Arachidonic Acid (Declerck et al., 2017) | Benzo(a)pyrene (Declerck et al., 2017) |
| 16 | Atrazine (Gunes et al., 2009) | Arsenic (Peyre et al., 2014) | Bisphenol A (Declerck et al., 2017) |
| 17 | Azacitidine (Gunes et al., 2009) | Arsenic Trioxide (Gunes et al., 2009) | Colforsin (Zhang et al., 2015) |
| 18 | Benzo(a)pyrene (Huang et al., 2009) | Asbestos (Zhang et al., 2015) | Copper Sulfate (Zhang et al., 2015) |
| 19 | Berberine (Hirai et al., 2019) | Benzene (Kuhara et al., 2009) | Deoxynivalenol (Samuelov et al., 2013) |
| 20 | Bis(tri-n-butyltin)oxide (Kuhara et al., 2009) | Benzimidazole (Pearen and Muscat, 2010) | Dexamethasone (Bongard et al., 2006) |
| 21 | Bisphenol A (Declerck et al., 2017) | Cadmium (Ariazi and Jordan, 2006) | Dorsomorphin (Kuhara et al., 2009) |
| 22 | Cadmium (Ariazi and Jordan, 2006) | Cadmium Chloride (Ariazi and Jordan, 2006) | Doxorubicin (Zhang et al., 2015) |
| 23 | Cadmium Sulfate (Ariazi and Jordan, 2006) | Cobaltous Chloride (Peyre et al., 2014) | Epigallocatechin gallate (Samuelov et al., 2013) |
| 24 | Calcitriol (Huang et al., 2009) | Cocaine (Ariazi and Jordan, 2006) | Exoenzyme C3, Clostridium botulinum (Peyre et al., 2014) |
| 25 | Cisplatin (Bongard et al., 2006) | Colforsin (Zhang et al., 2015) | Gemcitabine (Kuhara et al., 2009) |
| 26 | Colforsin (Zhang et al., 2015) | Copper Sulfate (Zhang et al., 2015) | Geranylgeranyl pyrophosphate (Peyre et al., 2014) |
| 27 | Copper (Ariazi and Jordan, 2006) | Curcumin (Peyre et al., 2014) | Ionomycin (Ariazi and Jordan, 2006) |
| 28 | Copper Sulfate (Zhang et al., 2015) | Dalcetrapib (Declerck et al., 2017) | Jinfukang (Fujiki et al., 2009) |
| 29 | Cyclosporine (Kuhara et al., 2009) | Dichloroethylene (Zhang et al., 2006) | Leflunomide (Declerck et al., 2017) |
| 30 | Cytosporone B (CsnB (Zhan et al., 2008) | Dinoprostone (Huang et al., 2009) | Methotrexate (Huang et al., 2009) |
| 31 | Daidzein (Huang et al., 2009) | Dorsomorphin (Declerck et al., 2017) | Mevalonic Acid (Declerck et al., 2017) |
| 32 | Dichloroethylene (Zhang et al., 2006) | Doxorubicin (Zhang et al., 2015) | Mono-(2-ethylhexyl) phthalate (Kuhara et al., 2009) |
| 33 | Dicrotophos (Kuhara et al., 2009) | Epigallocatechin Gallate (Ariazi and Jordan, 2006) | Nickel (Fujiki et al., 2009) |
| 34 | Dihydrotestosterone (Ariazi and Jordan, 2006) | Estradiol (Peyre et al., 2014) | PCI 5002 (Zhang et al., 2015) |
| 35 | Doxorubicin (Zhang et al., 2015) | Exoenzyme C3, Clostridium botulinum (Peyre et al., 2014) | Potassium chromate(VI) (Kuhara et al., 2009) |
| 36 | Epigallocatechin Gallate (Kuhara et al., 2009) | Geraniol (Ariazi and Jordan, 2006) | Prednisolone (Declerck et al., 2017) |
| 37 | Estradiol (Huang et al., 2009) | Hydroquinone (Gunes et al., 2009) | Prostaglandin A2 (Zhu et al., 2013) |
| 38 | Ethyl[2,3,4-trimethoxy-6-(i-oc-tanoyl)phenyl] acetate (TMPA) (Zhan et al., 2008) | Indomethacin (Declerck et al., 2017) | Raloxifene Hydrochloride (Huang et al., 2009) |
| 39 | Fenretinide (Huang et al., 2009) | Isoxazolopyridinone (Pearen and Muscat, 2010) | Silicon Dioxide (Zhang et al., 2015) |
| 40 | Fluorouracil (Ariazi and Jordan, 2006) | Lactic Acid (Huang et al., 2009) | Simvastatin (Ariazi and Jordan, 2006) |
| 41 | Fulvestrant (Zhang et al., 2006) | Medroxyprogesterone Acetate (Ariazi and Jordan, 2006) | Sodium Arsenite (Kuhara et al., 2009) |
| 42 | Genistein (Declerck et al., 2017) | Methotrexate (Ariazi and Jordan, 2006) | Sulforafan (Zhang et al., 2006) |
| 43 | Glutathione (Gunes et al., 2009) | Methylmercuric Chloride (Kuhara et al., 2009) | Tamoxifen (Kuhara et al., 2009) |
| 44 | Glycitein (Ariazi and Jordan, 2006) | MLN7243 (Ariazi and Jordan, 2006) | Tetradecanoylphorbol Acetate (Fujiki et al., 2009) |
| 45 | Hydrogen Peroxide (Bongard et al., 2006) | Mono-(2-ethylhexyl)phthalate (Samuelov et al., 2013) | Torcetrapib (Huang et al., 2009) |
| 46 | Indomethacin (Declerck et al., 2017) | N-(2-(4-bromocinn-amylamino)ethyl)-5-isoquinolinesulfonam-ide (Samuelov et al., 2013) | Tretinoin (Fujiki et al., 2009) |
| 47 | Jinfukang (Fujiki et al., 2009) | Nickel (Fujiki et al., 2009) | Urethane (Fujiki et al., 2009) |
| 48 | Lactic Acid (Ariazi and Jordan, 2006) | Nickel Sulfate (Fujiki et al., 2009) | Valproic Acid (Zhang et al., 2006) |
| 49 | Leflunomide (Huang et al., 2009) | Paricalcitol (Ariazi and Jordan, 2006) | Y 27632 (Ariazi and Jordan, 2006) |
| 50 | Leptomycin B (Gunes et al., 2009) | PCI 5002 (Zhang et al., 2015) |  |
| 51 | Methylmercuric chloride (Ariazi and Jordan, 2006) | Pentabromodiphenyl Ether (Samuelov et al., 2013) |  |
| 52 | Mono-(2-ethylhexyl)phthalate (Samuelov et al., 2013) | Phosphorus (Ariazi and Jordan, 2006) |  |
| 53 | N-(2-(4-bromocin-namylamino)ethyl)-5-isoquinolinesulfonam-ide (Samuelov et al., 2013) | Pirinixic Acid (Bongard et al., 2006) |  |
| 54 | Nickel Sulfate (Fujiki et al., 2009) | Polychlorinated Biphenyls (Peyre et al., 2014) |  |
| 55 | N-Nitrosopyrrolidine (Huang et al., 2009) | Potassium chromate(VI) (Samuelov et al., 2013) |  |
| 56 | Paricalcitol (Declerck et al., 2017) | Proanthocyanidins (Zhang et al., 2015) |  |
| 57 | PCI 5002 (Zhang et al., 2015) | Propionaldehyde (Zhang et al., 2006) |  |
| 58 | Pentabromodiphenyl Ether (Peyre et al., 2014) | Rotenone (Peyre et al., 2014) |  |
| 59 | Pesticides (Gunes et al., 2009) | Silicon Dioxide (Zhang et al., 2015) |  |
| 60 | Phosphorus (Declerck et al., 2017) | Sodium Arsenite (Declerck et al., 2017) |  |
| 61 | Potassium Chromate(VI) (Samuelov et al., 2013) | Sodium Dodecyl Sulfate (Peyre et al., 2014) |  |
| 62 | Proanthocyanidins (Zhang et al., 2015) | Sodium Selenite (Declerck et al., 2017) |  |
| 63 | Progesterone (Ariazi and Jordan, 2006) | Tetrachlorodibenzodioxin (Samuelov et al., 2013) |  |
| 64 | Quercetin (Samuelov et al., 2013) | Tetradecanoylphorbol Acetate (Samuelov et al., 2013) |  |
| 65 | Raloxifene Hydrochloride (Declerck et al., 2017) | Thalidomide (Zhang et al., 2015) |  |
| 66 | Silicon Dioxide (Zhang et al., 2015) | Thapsigargin (Samuelov et al., 2013) |  |
| 67 | Todium Arsenite (Zhang et al., 2006) | Torcetrapib (Gunes et al., 2009) |  |
| 68 | T-2 Toxin (Gunes et al., 2009) | Tretinoin (Declerck et al., 2017) |  |
| 69 | Tert-Butylhydroperoxide (Kuhara et al., 2009) | Trichostatin A (Declerck et al., 2017) |  |
| 70 | Testosterone (Ariazi and Jordan, 2006) | Urethane (Bongard et al., 2006) |  |
| 71 | Tetrachlorodibenzodioxin (Kuhara et al., 2009) | Valproic Acid (Kuhara et al., 2009) |  |
| 72 | Tetradecanoylphorbol Acetate (Fujiki et al., 2009) |  |  |
| 73 | Thalidomide (Zhang et al., 2015) |  |  |
| 74 | Torcetrapib (Zhang et al., 2006) |  |  |
| 75 | Tretinoin (Fujiki et al., 2009) |  |  |
| 76 | Trichostatin A (Gunes et al., 2009) |  |  |
| 77 | Tris(2-butoxyethyl) phosphate (Ariazi and Jordan, 2006) |  |  |
| 78 | Urethane (Fujiki et al., 2009) |  |  |
| 79 | Vitamin K 3 (Samuelov et al., 2013) |  |  |

# Reference

Ariazi, E.A., and Jordan, V.C. (2006). Estrogen-related receptors as emerging targets in cancer and metabolic disorders. *Curr Top Med Chem.* 6**,** 203-215. doi:10.2174/1568026610606030203

Bongard, V., Marc, D., Philippe, V., Jean-Louis, M., and Maryse, L.M. (2006). Incidence rate of adverse drug reactions during long-term follow-up of patients newly treated with amiodarone. *Am J Ther.* 13**,** 315-319. doi:10.1097/00045391-200607000-00007

Declerck, K., Remy, S., Wohlfahrt-Veje, C., Main, K.M., Van Camp, G., Schoeters, G., et al. (2017). Interaction between prenatal pesticide exposure and a common polymorphism in the PON1 gene on DNA methylation in genes associated with cardio-metabolic disease risk-an exploratory study. *Clin Epigenetics.* 9**,** 35. doi:10.1186/s13148-017-0336-4

Fujiki, K., Kano, F., Shiota, K., and Murata, M. (2009). Expression of the peroxisome proliferator activated receptor gamma gene is repressed by DNA methylation in visceral adipose tissue of mouse models of diabetes. *BMC Biol.* 7**,** 38. doi:10.1186/1741-7007-7-38

Gunes, A., Melkersson, K.I., Scordo, M.G., and Dahl, M.L. (2009). Association between HTR2C and HTR2A polymorphisms and metabolic abnormalities in patients treated with olanzapine or clozapine. *J Clin Psychopharmacol.* 29**,** 65-68. doi:10.1097/JCP.0b013e31819302c3

Hirai, T., Mitani, Y., Kurumisawa, K., Nomura, K., Wang, W., Nakashima, K.I., et al. (2019). Berberine stimulates fibroblast growth factor 21 by modulating the molecular clock component brain and muscle Arnt-like 1 in brown adipose tissue. *Biochem Pharmacol.* 164**,** 165-176. doi:10.1016/j.bcp.2019.04.017

Huang, R.C., Mori, T.A., Burke, V., Newnham, J., Stanley, F.J., Landau, L.I., et al. (2009). Synergy between adiposity, insulin resistance, metabolic risk factors, and inflammation in adolescents. *Diabetes Care.* 32**,** 695-701. doi:10.2337/dc08-1917

Inamoto, T., Papineni, S., Chintharlapalli, S., Cho, S.D., Safe, S., and Kamat, A.M. (2008). 1,1-Bis(3'-indolyl)-1-(p-chlorophenyl)methane activates the orphan nuclear receptor Nurr1 and inhibits bladder cancer growth. *Mol Cancer Ther.* 7**,** 3825-3833. doi:10.1158/1535-7163.MCT-08-0730

Kuhara, T., Ohse, M., Inoue, Y., and Shinka, T. (2009). Five cases of beta-ureidopropionase deficiency detected by GC/MS analysis of urine metabolome. *J Mass Spectrom.* 44**,** 214-221. doi:10.1002/jms.1500

Ordentlich, P., Yan, Y., Zhou, S., and Heyman, R.A. (2003). Identification of the antineoplastic agent 6-mercaptopurine as an activator of the orphan nuclear hormone receptor Nurr1. *J Biol Chem.* 278**,** 24791-24799. doi:10.1074/jbc.M302167200

Pearen, M.A., and Muscat, G.E. (2010). Minireview: Nuclear hormone receptor 4A signaling: implications for metabolic disease. *Mol Endocrinol.* 24**,** 1891-1903. doi:10.1210/me.2010-0015

Peyre, L., Rouimi, P., De Sousa, G., Helies-Toussaint, C., Carre, B., Barcellini, S., et al. (2014). Comparative study of bisphenol A and its analogue bisphenol S on human hepatic cells: a focus on their potential involvement in nonalcoholic fatty liver disease. *Food Chem Toxicol.* 70**,** 9-18. doi:10.1016/j.fct.2014.04.011

Samuelov, L., Sarig, O., Harmon, R.M., Rapaport, D., Ishida-Yamamoto, A., Isakov, O., et al. (2013). Desmoglein 1 deficiency results in severe dermatitis, multiple allergies and metabolic wasting. *Nat Genet.* 45**,** 1244-1248. doi:10.1038/ng.2739

Zhan, Y., Du, X., Chen, H., Liu, J., Zhao, B., Huang, D., et al. (2008). Cytosporone B is an agonist for nuclear orphan receptor Nur77. *Nat Chem Biol.* 4**,** 548-556. doi:10.1038/nchembio.106

Zhang, J., Frerman, F.E., and Kim, J.J. (2006). Structure of electron transfer flavoprotein-ubiquinone oxidoreductase and electron transfer to the mitochondrial ubiquinone pool. *Proc Natl Acad Sci U S A.* 103**,** 16212-16217. doi:10.1073/pnas.0604567103

Zhang, L., Nichols, R.G., Correll, J., Murray, I.A., Tanaka, N., Smith, P.B., et al. (2015). Persistent Organic Pollutants Modify Gut Microbiota-Host Metabolic Homeostasis in Mice Through Aryl Hydrocarbon Receptor Activation. *Environ Health Perspect.* 123**,** 679-688. doi:10.1289/ehp.1409055

Zhu, X., Walton, R.G., Tian, L., Luo, N., Ho, S.R., Fu, Y., et al. (2013). Prostaglandin A2 enhances cellular insulin sensitivity via a mechanism that involves the orphan nuclear receptor NR4A3. *Horm Metab Res.* 45**,** 213-220. doi:10.1055/s-0032-1327619
